# Risk factors for acute kidney injury and its impact on 3-year mortality after thoracic endovascular repair for type B aortic dissection

**DOI:** 10.3389/fcvm.2025.1691995

**Published:** 2025-12-11

**Authors:** Na Li, Zhiqiang Zhang, Yasong Wang, Tienan Zhou, Xiaozeng Wang

**Affiliations:** State Key Laboratory of Frigid Zone Cardiovascular Disease, Cardiovascular Research Institute and Department of Cardiology, General Hospital of Northern Theater Command, Shenyang, Liaoning, China

**Keywords:** acute Stanford type B aortic dissection, thoracic endovascular aortic repair, acute kidney injury, risk factors of acute kidney injury, impact on 3-year mortality

## Abstract

**Objective:**

Acute kidney injury (AKI) following thoracic endovascular aortic repair (TEVAR) significantly impacts outcomes. This study aimed to identify independent predictors of AKI after TEVAR for acute Stanford type B aortic dissection (ATBAD) and evaluate its impact on long-term mortality.

**Methods:**

This retrospective analysis included 745 consecutive patients who underwent TEVAR for ATBAD at the General Hospital of Northern Theater Command between February 2004 and November 2022. Acute kidney injury was diagnosed based on a serum creatinine increase of ≥26.5 μmol/L within 48 h, a ≥1.5-fold increase from baseline within 7 days, or urine output <0.5 mL/kg/h for 6 h. Univariate and multivariate logistic regressions identified risk factors for AKI and all-cause mortality.

**Results:**

AKI occurred in 75 patients (10.1%). The patients who developed AKI had a higher baseline serum creatinine level (85.97 vs. 78.30 μmol/L; *P* = 0.009), received more contrast volume (246.13 ± 92.00 vs. 221.17 ± 69.92 mL; *P* = 0.042), and had a higher prevalence of bilateral renal artery involvement (14.3% vs. 5.1%; *P* = 0.020). After a mean follow-up of 36 months, the multivariate analysis identified the following three independent predictors: bilateral renal artery involvement [odds ratio (OR): 4.381, 95% confidence interval (CI): 1.54–12.45; *P* = 0.006], serum creatinine >114 μmol/L (OR: 2.86, 95% CI: 1.38–5.93; *P* = 0.005), and contrast volume >290 mL (OR: 2.36, 95% CI: 1.10–5.09; *P* = 0.028). All-cause mortality was significantly higher in the AKI group (16.1% vs. 4.4%; *P* < 0.001). AKI remained an independent predictor of mortality (OR: 3.02, 95% CI: 1.46–6.28; *P* = 0.003) after adjusting for age, baseline creatinine, and medication use.

**Conclusions:**

AKI remains highly prevalent in patients undergoing TEVAR for ATBAD. Bilateral renal artery involvement, elevated baseline creatinine, and contrast volume >290 mL independently predicted AKI. Development of AKI was associated with a threefold increase in long-term mortality, emphasizing the importance of risk stratification and nephroprotective strategies.

## Introduction

Acute Stanford type B aortic dissection (ATBAD) is a life-threatening cardiovascular emergency with rising global incidence (1). Thoracic endovascular aortic repair (TEVAR) has become the preferred treatment for complicated type B dissection, demonstrating superior outcomes compared with open surgery and optimal medical therapy (2, 3). The 2022 Society of Thoracic Surgeons/American Association for Thoracic Surgery (STS/AATS) guidelines recommend TEVAR as the first-line therapy for complicated cases (4). Despite these advances, postoperative complications significantly impact outcomes.

Acute kidney injury (AKI) is among the most serious complications following TEVAR, with an incidence ranging from 14% to 30.8% (5–7). The International Registry of Acute Aortic Dissection reported an AKI incidence of 16.2% among 630 patients who underwent TEVAR for acute type B dissection (5). The development of AKI increases in-hospital mortality from 4% to 18.6% and reduces 5-year survival from 80% to 51% (5, 8). Post-TEVAR AKI pathophysiology involves contrast-induced nephropathy, renal malperfusion from dissection flap involvement, atheroembolic disease, and hemodynamic instability (9). Pre-existing renal dysfunction increases a patient’s susceptibility to perioperative insults (6).

Studies that specifically address AKI after TEVAR for acute type B dissection are scarce. Most studies combine heterogeneous populations or include chronic dissections, potentially obscuring unique risk factors (10, 11). Anatomical factors, particularly bilateral renal artery (RA) involvement, require further evaluation. Recent research demonstrated that the risk of AKI increased by 5% per 10 mL of contrast used in endovascular aortic repair, but TEVAR-specific thresholds remain undefined (12). The ongoing CULTURE trial is investigating contrast dilution strategies (13).

Therefore, this study aimed to identify independent predictors of AKI in patients undergoing TEVAR for acute Stanford type B aortic dissection, emphasizing modifiable procedural factors and anatomical characteristics for risk stratification, and to determine AKI's impact on long-term survival.

## Methods

### Data sources and population

This retrospective cohort study analyzed a single-center registry of consecutive patients with ATBAD who underwent thoracic endovascular aortic repair at the General Hospital of Northern Theater Command. From February 2004 to November 2022, all patients diagnosed with acute type B aortic dissection by computed tomography angiography and treated with TEVAR were enrolled in the institutional database. The study protocol received approval from the hospital's research ethics committee with a waiver of informed consent due to its retrospective nature, and the investigation adhered to the principles of the Declaration of Helsinki.

The inclusion criteria comprised patients aged 18 years or older with confirmed ATBAD (symptom onset within 14 days) who underwent TEVAR as the primary intervention and had complete renal function laboratory data for assessment of postoperative acute kidney injury. The exclusion criteria encompassed concomitant aortic aneurysm, traumatic aortic injury, penetrating aortic ulcers, and insufficient pre- and post-procedural renal function documentation.

The collected clinical data included demographics, comorbidities, laboratory values, procedural characteristics, and outcomes through standardized case report forms maintained in the departmental database.

### Description of procedure

The patients included in this study were examined using computed tomography angiography (CTA) and 3D reconstructions to evaluate the nature, location, and extent of the disease, as well as the involvement of the branches, among other factors. All the patients were admitted to the cardiovascular intensive care unit and received appropriate treatments, such as oxygen inhalation and blood pressure (BP) and heart rate control. Prompt TEVAR was performed for patients with unrelieved severe chest pain or compromised organ perfusion due to ATBAD. Selective TEVAR treatment was administered to the remaining patients once their condition stabilized. All the angiographic procedures used isotonic, non-ionic contrast agents, which were injected into the artery at a fixed concentration. Images were acquired during the arterial phase. Fusion imaging based on preoperative CTA was preferred to optimize navigation. All the operations were performed by a team of experienced experts using standardized equipment platforms.

### Definitions and outcomes

According to the Kidney Disease: Improving Global Outcomes (KDIGO) clinical practice guidelines, AKI is defined as meeting any of the following criteria: (1) an absolute increase in serum creatinine (SCr) level of ≥0.3 mg/dL (≥26.5 µmol/L) within 48 h; (2) an increase in SCr level of at least 50% from a known or presumed baseline within 7 days; or (3) urine output of less than 0.5 mL/kg/h for more than 6 h (14). All-cause mortality encompassed death from any cause during follow-up. Cause-specific mortality was classified as aorta-related death (aortic rupture, malperfusion syndrome, or retrograde dissection complications) or cardiac death (myocardial infarction, heart failure, or sudden cardiac death). Composite aortic events included recurrent aortic dissection, endoleak, and distal stent-induced new entry (dSINE). Recurrent aortic dissection was defined as a new dissection in previously unaffected segments or extension beyond the treated segment, confirmed by computed tomography angiography. Endoleak was classified by standard criteria (types I–IV), with only persistent endoleaks lasting more than 30 days or those requiring intervention included. Distal stent-induced new entry was defined as a new intimal tear at the stent-graft distal edge, confirmed by imaging. Reintervention comprised any secondary endovascular or open surgical procedure addressing complications from the initial TEVAR or disease progression. Planned staged procedures were excluded unless performed earlier due to complications.

Renal artery involvement was assessed from preoperative computed tomography angiography and categorized as none (both arteries from the true lumen), unilateral (dissection flap in one artery), or bilateral (dissection flap in both arteries).

### Follow-up and data collection

Follow-up included direct patient contact, telephone interview, and referring physician contact to evaluate clinical outcomes. The clinical outcomes included mortality and aortic events in the early and late periods. Follow-up was performed in the 1st, 3rd, 6th, 12th, 24th, and 36th month.

### Statistical analysis

Continuous variables are presented as mean ± standard deviation for normally distributed data or median with interquartile range for non-normally distributed data and were assessed using the Shapiro–Wilk test. Categorical variables are expressed as frequencies and percentages. Comparisons between the AKI and non-AKI groups employed Student's *t*-test or the Mann–Whitney *U* test for continuous variables and the chi-square or Fisher's exact test for categorical variables as appropriate. For continuous variables, the optimal cut-off value for predicting AKI was determined through receiver operating characteristic (ROC) curve analysis and Youden's index, which was then transformed into a dichotomous variable and incorporated into the final model. The univariate logistic regression analysis identified potential predictors of AKI, with *P* < 0.05 considered significant for inclusion in multivariate analysis. Multivariate logistic regression using backward stepwise selection (removal criterion *P* > 0.10) determined the independent predictors. A Kaplan–Meier survival analysis with the log-rank test compared the long-term outcomes between the groups. Follow-up duration was calculated from the date of TEVAR to the date of death or last contact. Patients lost to follow-up were censored at the date of last contact. All the analyses were performed using SPSS version 26.0 (IBM Corp, Armonk, NY, USA) and R 4.2.3 statistical software, with two-sided *P* < 0.05 considered statistically significant.

## Results

### Population characteristics

Among 745 patients who underwent thoracic endovascular aortic repair for acute Stanford type B aortic dissection, 75 patients (10.1%) developed postoperative AKI, according to the KDIGO criteria.

Table 1 shows the baseline characteristics of both groups. The patients who developed AKI had higher systolic blood pressure (SBP; 165.64 ± 25.06 vs. 156.94 ± 25.04 mmHg; *P* = 0.004), higher diastolic blood pressure (DBP; 93.92 ± 19.08 vs. 89.42 ± 16.65 mmHg; *P* = 0.029), and a higher prevalence of chronic renal failure (6.67% vs. 1.19%; *P* = 0.003). Baseline serum creatinine was significantly elevated in the AKI group [85.97 [68.29–120.25] vs. 78.30 [66.00–95.01] μmol/L; *P* = 0.009]. Post-TEVAR serum creatinine was markedly higher in the AKI group [122.00 [103.25–184.00] vs. 79.00 [66.00–94.00] μmol/L; *P* < 0.001].

**Table 1:** Baseline characteristics and procedural variables of the patients with and without acute kidney injury.

Variable	AKI (*n* = 75)	Non-AKI (*n* = 670)	*P*-value
Demographics			
Age, years	53.56 ± 12.39	53.44 ± 11.42	0.935
Male sex, %	64 (85.3%)	536 (80.0%)	0.269
Body mass index, kg/m^2^	26.70 ± 4.10	26.42 ± 3.94	0.615
Clinical presentation			
Chest or back pain, %	69 (92.0%)	611 (91.2%)	0.815
Systolic blood pressure, mmHg	165.64 ± 25.06	156.94 ± 25.04	0.004
Diastolic blood pressure, mmHg	93.92 ± 19.08	89.42 ± 16.65	0.029
Heart rate, bpm	84.56 ± 14.05	84.06 ± 15.37	0.788
Medical history, %			
Hypertension	63 (84.0%)	556 (83.0%)	0.824
Coronary heart disease	10 (13.3%)	102 (15.2%)	0.664
Diabetes mellitus	3 (4.0%)	30 (4.5%)	1.000
Previous stroke	3 (4.0%)	59 (8.8%)	0.153
Chronic renal failure	5 (6.7%)	8 (1.2%)	0.003
Current smoking	40 (53.3%)	394 (58.8%)	0.362
Laboratory values			
Serum creatinine, μmol/L^a^	85.97 [68.29–120.25]	78.30 [66.00–95.01]	0.009
Post-TEVAR creatinine, μmol/L^a^	122.00 [103.25–184.00]	79.00 [66.00–94.00]	<0.001
Hemoglobin, g/L	132.03 ± 17.99	135.19 ± 16.74	0.125
White blood cell count, ×10^9^/L	11.20 ± 4.00	10.95 ± 3.26	0.539
Platelet count, ×10^9^/L^a^	169 [134–219]	175 [145–221]	0.305
Total cholesterol, mmol/L	4.28 ± 0.96	4.45 ± 1.85	0.456
LDL cholesterol, mmol/L	2.39 ± 0.75	2.44 ± 0.71	0.599
Blood glucose, mmol/L	6.55 ± 1.98	6.21 ± 1.44	0.108
In-hospital medications, %			
ACE inhibitors/ARBs	67 (89.3%)	539 (80.7%)	0.067
Calcium channel blockers	71 (94.7%)	627 (93.9%)	0.983
Beta-blockers	71 (94.7%)	640 (95.8%)	0.871
Diuretics	42 (56.0%)	181 (27.2%)	<0.001
Statins	31 (41.3%)	383 (57.3%)	0.008
Procedural characteristics			
Contrast volume, mL	246.13 ± 92.00	221.17 ± 69.92	0.042
Stent length, mm	150.34 ± 33.11	161.05 ± 29.28	0.003
Stent manufacturer			0.614
Cook	28 (37.8%)	290 (43.5%)	
Medtronic	20 (27.0%)	172 (25.8%)	
Micro Port	26 (35.1%)	204 (30.6%)	
Anatomical features, %			
Renal artery involvement			0.020
None	19 (38.8%)	251 (50.8%)	
Unilateral	23 (46.9%)	218 (44.1%)	
Bilateral	7 (14.3%)	25 (5.1%)	
Pleural effusion	17 (22.7%)	118 (17.7%)	0.287
Pericardial effusion	1 (1.3%)	28 (4.2%)	0.369

ACE, angiotensin-converting enzyme; ARB, angiotensin receptor blocker; LDL, low-density lipoprotein; TEVAR, thoracic endovascular aortic repair.

All other continuous variables are presented as mean ± standard deviation. Categorical variables presented as *n* (%).

a Data presented as median [interquartile range].

Diuretic use during hospitalization was more frequent in the AKI group (56.00% vs. 27.18%; *P* < 0.001), while statin use was less common (41.33% vs. 57.34%; *P* = 0.008). The AKI group received a higher contrast volume (246.13 ± 92.00 vs. 221.17 ± 69.92 mL; *P* = 0.042), despite having a shorter stent length (150.34 ± 33.11 vs. 161.05 ± 29.28 mm; *P* = 0.003).

### Independent predictors of acute kidney injury

The univariate analysis identified the following factors to be associated with AKI: current drinking [odds ratio (OR): 0.549, 95% confidence interval (CI): 0.328–0.918, *P* = 0.022], diuretic use (OR: 3.410, 95% CI: 2.096–5.549, *P* < 0.001), statin use (OR: 0.524, 95% CI: 0.323–0.851, *P* = 0.009), serum creatinine >114 μmol/L (OR: 3.441, 95% CI: 2.023–5.854, *P* < 0.001), systolic blood pressure >173 mmHg (OR: 1.957, 95% CI: 1.190–3.219, *P* = 0.008), bilateral renal artery involvement (OR: 3.699, 95% CI: 1.418–9.652, *P* = 0.008), stent length >124 mm (OR: 0.372, 95% CI: 0.202–0.686, *P* = 0.002), and contrast volume >290 mL (OR: 2.931, 95% CI: 1.625–5.284, *P* < 0.001) (Table 2).

**Table 2:** Univariate and multivariate analyses of risk factors for acute kidney injury.

Variable	Univariate analysis		Multivariate analysis	
	Crude OR (95% CI)	*P*-value	Adjusted OR (95% CI)	*P*-value
Current drinking	0.549 (0.328–0.918)	0.022	—	—
Diuretic use	3.410 (2.096–5.549)	<0.001	—	—
Statin use	0.524 (0.323–0.851)	0.009	—	—
Serum creatinine (per μmol/L)	1.003 (1.001–1.006)	0.012	—	—
Serum creatinine >114 μmol/L	3.441 (2.023–5.854)	<0.001	2.856 (1.375–5.931)	0.005
Systolic blood pressure (per mmHg)	1.013 (1.004–1.023)	0.005	—	—
Systolic blood pressure >173 mmHg	1.957 (1.190–3.219)	0.008	—	—
Renal artery involvement				
None	1.000 (reference)	—	1.000 (reference)	—
Unilateral	1.394 (0.739–2.628)	0.305	1.542 (0.767–3.097)	0.224
Bilateral	3.699 (1.418–9.652)	0.008	4.381 (1.543–12.445)	0.006
Stent length (per mm)	0.986 (0.978–0.995)	0.001	—	—
Stent length >124 mm	0.372 (0.202–0.686)	0.002	—	—
Contrast volume (per mL)	1.004 (1.001–1.006)	0.014	—	—
Contrast volume >290 mL	2.931 (1.625–5.284)	<0.001	2.362 (1.096–5.092)	0.028

CI, confidence interval.

Multivariate model C-statistic = 0.742. Variables with *P* < 0.05 in the univariate analysis were included in the multivariate model. Dashes indicate variables not included in the final multivariate model.

The multivariate logistic regression analysis identified the following three independent predictors of AKI: bilateral renal artery involvement (adjusted OR: 4.381, 95% CI: 1.543–12.445, *P* = 0.006), serum creatinine >114 μmol/L (adjusted OR: 2.856, 95% CI: 1.375–5.931, *P* = 0.005), and contrast volume >290 mL (adjusted OR: 2.362, 95% CI: 1.096–5.092, *P* = 0.028).

### Clinical outcomes stratified by the presence or absence of AKI

**Figure 1:**
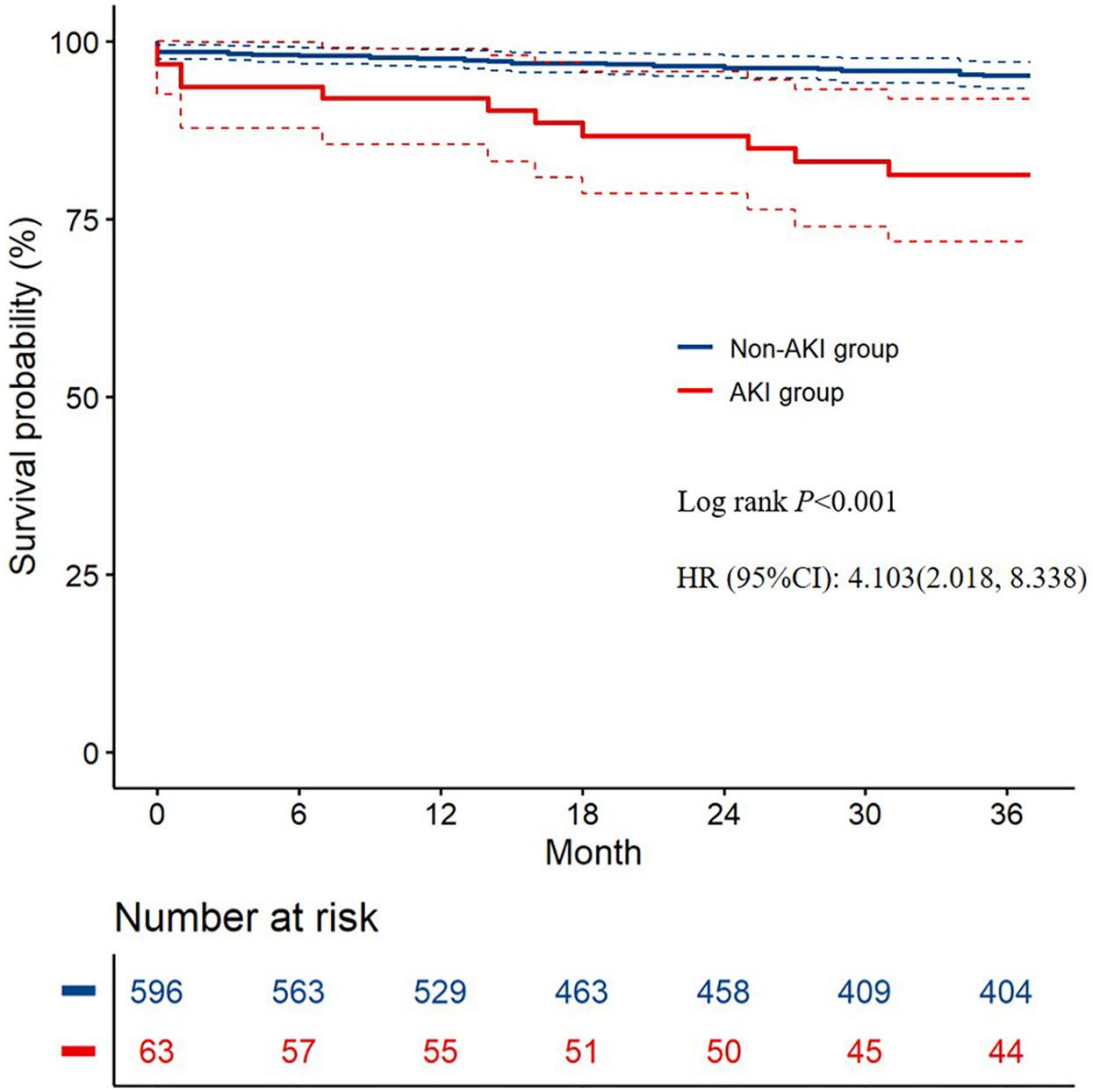
Kaplan–Meier curve analysis of all-cause mortality in the two groups.

At a mean follow-up of 36 months, data were available for 64 patients (85.3%) in the AKI group and 602 patients (89.9%) in the non-AKI group. All-cause mortality was significantly higher in the AKI group (16.1% vs. 4.36%; *P* < 0.001) (Figure 1). Cardiac death occurred more frequently in the AKI group (3.23% vs. 0.34%; *P* = 0.046), while aorta-related death showed no significant difference (4.84% vs. 2.51%; *P* = 0.509).

Composite aortic events occurred in 14.52% of cases in the AKI group vs. 8.71% in the non-AKI group (*P* = 0.133), including recurrent aortic dissection (9.68% vs. 4.69%; *P* = 0.165), endoleak (8.06% vs. 4.52%; *P* = 0.355), and distal SINE (0.00% vs. 0.84%; *P* = 1.000). The reintervention rates were similar between the groups (1.61% vs. 2.18%; *P* = 1.000) (Table 3).

**Table 3:** Clinical outcomes during 36 months of follow-up stratified by the presence or absence of acute kidney injury.

Clinical outcomes	AKI (*n* = 64)	Non-AKI (*n* = 602)	*P*-value
Mortality			
All-cause death	10 (16.1%)	26 (4.4%)	<0.001
Aorta-related death	3 (4.8%)	15 (2.5%)	0.509
Cardiac death	2 (3.2%)	2 (0.3%)	0.046
Aortic events			
Composite aortic events^a^	9 (14.5%)	52 (8.7%)	0.133
Recurrent aortic dissection	6 (9.7%)	28 (4.7%)	0.165
Endoleak	5 (8.1%)	27 (4.5%)	0.355
Distal SINE	0 (0.0%)	5 (0.8%)	1.000
Reintervention	1 (1.6%)	13 (2.2%)	1.000

a Composite of recurrent aortic dissection, endoleak, and dSINE.

### Univariate and multivariate analyses of risk factors for all-cause mortality

The univariate analysis identified seven significant predictors of all-cause mortality (Table 4). In the multivariate analysis, the following five factors remained independently associated with mortality: AKI (adjusted OR: 3.022, 95% CI: 1.455–6.278; *P* = 0.003), age (adjusted OR: 1.053, 95% CI: 1.023–1.085; *P* < 0.001), use of β-blockers (adjusted OR: 0.239, 95% CI: 0.090–0.640; *P* = 0.004), use of statins (adjusted OR: 0.456, 95% CI: 0.226–0.922; *P* = 0.029), and serum creatinine (adjusted OR: 1.002, 95% CI: 1.001–1.003; *P* < 0.001).

**Table 4:** Univariate and multivariate analyses of risk factors for all-cause mortality.

Variable	Univariate analysis		Multivariate analysis	
	Odds ratio (95% CI)	*P*-value	Adjusted odds ratio (95% CI)	*P*-value
Acute kidney injury	4.104 (2.019–8.341)	<0.001	3.022 (1.455–6.278)	0.003
Age (per year increasing)	1.049 (1.018–1.080)	0.002	1.053 (1.023–1.085)	<0.001
Chronic renal failure	5.488 (1.683–17.897)	0.005	—	—
Beta-blockers	0.232 (0.090–0.598)	0.002	0.239 (0.090–0.640)	0.004
Statins	0.443 (0.221–0.886)	0.021	0.456 (0.226–0.922)	0.029
Hemoglobin (per g/L)	0.975 (0.964–0.987)	<0.001	—	—
Serum creatinine (per μmol/L)	1.002 (1.001–1.003)	<0.001	1.002 (1.001–1.003)	<0.001

CI, confidence interval.

Variables with *P* < 0.05 in the univariate analysis were included in the multivariate model. Dashes indicate variables not included in the final multivariate model.

### Sensitivity analyses

To test the robustness of the primary findings, sensitivity analyses were performed. After excluding the patients with pre-existing chronic renal failure (*n* = 14), bilateral renal artery involvement [adjusted OR (aOR): 4.11, 95% CI: 1.43–11.87; *P* = 0.009], serum creatinine >114 µmol/L (aOR: 2.74, 95% CI: 1.31–5.75; *P* = 0.007), and contrast volume >290 mL (aOR: 2.42, 95% CI: 1.11–5.24; *P* = 0.026) remained independent predictors of AKI. Similarly, after excluding the patients with bilateral renal artery involvement (*n* = 42), serum creatinine >114 µmol/L (aOR: 3.02, 95% CI: 1.44–6.35; *P* = 0.004) and contrast volume >290 mL (aOR: 2.60, 95% CI: 1.18–5.72; *P* = 0.017) were still significantly associated with AKI. These analyses confirmed the stability of the identified risk factors.

## Discussion

AKI is a well-recognized complication after TEVAR and is associated with long-term sequelae, including chronic kidney disease (CKD) and increased mortality (15). The reported incidence of thoracic aortic disease after TEVAR ranges from 1.5% to 34% (16, 17) and is as high as 17.9%–52% in some series (18). In the present ATBAD cohort, AKI occurred in 10.07% of patients, consistent with these ranges. Notably, AKI significantly increases both early and late mortality independently of other risk factors, and this adverse effect persists even when renal function returns to baseline at discharge (19).

Elevated BP at admission has been repeatedly linked to AKI risk after TEVAR in ATBAD (20, 21). Luo et al. found that SBP >140 mmHg (OR: 2.288, 95% CI: 1.319–3.969) was an independent predictor (11), and An et al. showed that each 10 mmHg increase in DBP increased AKI risk 1.4-fold (OR: 1.418, 95% CI: 1.070–1.879) (10). High BP may reflect RA involvement and activation of the renin-angiotensin-aldosterone system (RAAS), and can exacerbate false lumen dilation, worsening renal ischemia (21). In our study, the univariate analysis indicated that SBP >173 mmHg significantly increased AKI incidence, aligning with previous reports.

Baseline renal dysfunction is another established risk factor. Multiple studies have shown that elevated preoperative SCr correlates with higher AKI rates post-TEVAR (22, 23). In our cohort, SCr >114 μmol/L was associated with increased AKI risk, likely reflecting reduced renal reserve and heightened susceptibility to ischemic or contrast-induced injury.

Contrast exposure is a modifiable intraoperative factor. A contrast volume >290 mL independently predicted AKI, and the AKI group received significantly higher doses than the non-AKI group. Prior evidence indicates that large, rapidly administered contrast loads can cause medullary ischemia, tubular epithelial degeneration, and oxidative stress (24). Although complex ATBAD anatomy, especially that with multiple intimal tears, often requires extensive imaging, limiting contrast volume through pre-procedural planning, fusion imaging, or intravascular ultrasound should be prioritized.

Bilateral RA involvement significantly increased AKI risk, whereas unilateral involvement did not. Potential mechanisms include the following: (1) reduced baseline perfusion when both RAs are supplied by the false lumen, with post-TEVAR true lumen expansion compressing the false lumen and further decreasing flow; (2) preserved contralateral perfusion in unilateral involvement, leading to compensation; and (3) higher baseline SCr in bilateral cases, with contrast exposure exacerbating injury (25).

Interestingly, current alcohol consumption was associated with reduced AKI risk. However, this association may be influenced by unmeasured lifestyle factors (e.g., diet, physical activity) or other comorbidities that correlate with alcohol consumption. Prior findings are mixed: a large Dutch cohort reported an inverse association with incident CKD (26) and Chinese national survey data showed higher CKD prevalence among non-drinkers (27). As our analysis focused on contrast-related AKI, the alcohol association should be interpreted cautiously and confirmed in targeted studies.

In summary, bilateral RA involvement, SCr >114 μmol/L, and contrast volume >290 mL are independent predictors of AKI after TEVAR for ATBAD. Identifying these factors preoperatively may enable risk stratification, guide perioperative optimization, and support contrast-sparing strategies to protect renal function.

This study had several limitations. First, it was a single-center retrospective analysis, which may limit generalizability and introduce unmeasured confounding. Second, preoperative SCr was only measured once, which may not reflect baseline renal function variability. Third, follow-up data for some clinical events relied on patient or family recall, introducing potential recall bias. Fourth, RA involvement was defined anatomically without direct hemodynamic or functional assessment, possibly underestimating the impact of unilateral lesions. Finally, long-term renal outcomes, including CKD progression, were not evaluated. Future multicenter prospective studies incorporating standardized functional imaging and long-term follow-up are warranted to validate these findings.

## Conclusions

AKI is a frequent and clinically significant complication after TEVAR for ATBAD, contributing to increased perioperative and long-term mortality. In this study, bilateral RA involvement, baseline SCr >114 μmol/L, and contrast volume >290 mL were identified as independent predictors. In addition, current alcohol consumption was associated with reduced AKI risk, though the cause remains uncertain. Recognition of these readily assessable factors will enable early risk stratification, targeted perioperative optimization, and the adoption of contrast-sparing techniques to minimize renal injury in high-risk patients.

## Data Availability

The original contributions presented in the study are included in the article/Supplementary Material, further inquiries can be directed to the corresponding author.
